# Evaluation of Prophylactic Defibrotide Use in Pediatric Hematopoietic Stem Cell Transplant Recipients: A Multicenter Retrospective Cohort Study

**DOI:** 10.3390/jpm16070368

**Published:** 2026-07-07

**Authors:** Archana Ramgopal, Tsuyoshi Fujita, Breana K. Goscicki, Shiva Sridar, Daniel Klein, Li Wang, Ramasubramanian Kalpatthi, Jignesh Dalal

**Affiliations:** 1 Division of Hematology, Oncology, and Stem Cell Transplantation, UPMC Children’s Hospital of Pittsburgh, University of Pittsburgh School of Medicine, Pittsburgh, PA 15224, USA; 2 Tufts University School of Medicine, Boston, MA 02111, USA; 3 Division of Hematology and Bone Marrow Transplant, Rainbow Babies & Children’s Hospital, Cleveland, OH 44106, USA

**Keywords:** defibrotide prophylaxis, sinusoidal obstruction syndrome, veno-occlusive disease, pediatric hematopoietic stem cell transplant

## Abstract

**Background/Objectives**: Sinusoidal obstruction syndrome (SOS), also known as veno-occlusive disease, is a serious complication of hematopoietic stem cell transplantation (HSCT), particularly in high and very high-risk pediatric patients. Despite known risk factors, U.S. data remain limited. The benefit of prophylactic defibrotide is uncertain, with prior trials yielding inconclusive results. This study evaluates its use and association with SOS incidence, healthcare burden, and outcomes. **Methods**: We performed a retrospective cohort study of 10,250 pediatric HSCT encounters using the Pediatric Health Information System. Patients were stratified as high-risk (n = 9584) or very high-risk (n = 666) per HARMONY criteria. Prophylactic defibrotide was used in 344 encounters; 9906 received none (therapeutic use allowed after SOS diagnosis). Outcomes included SOS incidence, length of stay (LOS), ICU admission, mortality, acute GVHD, and costs. Mixed-effects logistic regression models with patients as a random intercept were used (*p* < 0.05). **Results**: SOS incidence differences between the defibrotide prophylaxis and non-prophylaxis groups were not statistically significant in either risk category (high-risk: 26% [n = 79/302] vs. 7.1% [n = 663/9282] *p* = 0.495, OR 1.457, 95% CI 0.494–4.296; very high-risk: 31.0% [n = 13/42] vs. 21.6% [n = 135/624], *p* = 0.970, OR 1.081, 95% CI 0.019–60.769). The extremely wide confidence intervals indicate that the data are consistent with both benefit and harm of prophylaxis. Median LOS was longer in the prophylactic group (40 vs. 33 days, *p* < 0.001; 56 vs. 49 days, *p* = 0.296, respectively). ICU admissions (50.7% vs. 32.8%; 69.0% vs. 50.8%), mortality (7.9% vs. 3.5%; 23.8% vs. 10.9%), and costs ($443,537 vs. $205,325; *p* < 0.001) were also higher in the prophylaxis group. Acute GVHD incidence differences were not statistically significant, with contradictory directions between risk subgroups. **Conclusions**: Prophylactic defibrotide was not associated with reduced SOS incidence and was associated with higher ICU use, longer LOS, increased mortality, and greater costs. These findings represent associations, not causation, and likely reflect residual confounding by indication—as defibrotide prophylaxis was preferentially administered to patients perceived to be at highest clinical risk. Prospective studies with appropriate confounding adjustment are needed to clarify the role of defibrotide in SOS prevention.

## 1. Introduction

Sinusoidal obstruction syndrome (SOS), also known as veno-occlusive disease (VOD), is a severe complication of hematopoietic stem cell transplantation (HSCT), particularly prevalent in patients with defined risk factors. This complication occurs due to damage to the sinusoidal endothelial cells and hepatocytes, primarily from the conditioning regimen, but can include other factors that transpire in the HSCT process, including cytokine production, engraftment, and additional medications leading to injury [1]. SOS can range in severity and has the potential to lead to multi-organ dysfunction or failure. The likelihood of a patient developing SOS is based on various risk factors which can be considered transplant-related/modifiable or patient-related/non-modifiable. Transplant-related risk factors previously reported include myeloablative conditioning regimens, allogeneic HSCT, an unrelated or HLA-mismatched graft, and prior HSCT. Patient-related risk factors include underlying conditions such as osteopetrosis, thalassemia, or certain inherited syndromes as well as age ≤ 2 years, previous exposure to gemtuzumab or inotuzumab, and pre-existing liver dysfunction [1,2].

Ursodeoxycholic acid is the only widely supported agent that has been found to provide prophylactic benefit for the development of SOS [3]. However, this agent can present some logistical challenges for pediatric patients during periods of emetic risk and mucositis, as it is only available orally. Defibrotide is FDA-approved for the treatment of adult and pediatric patients with SOS, with renal or pulmonary dysfunction following hematopoietic stem-cell transplantation (HSCT) [4]. Defibrotide’s mechanism of action is multifactorial, including endothelial cell protection, in addition to anti-thrombotic, anti-inflammatory, and antioxidant properties. A few studies have been performed to assess the use of defibrotide as a prophylactic agent for SOS, but this has remained clinically controversial due to the discordance among study results. Corbacioglu et al. reported a reduction in the incidence of SOS in an open-label, multi-center, randomized controlled trial; however, the most recent HARMONY trial by Grupp et al. found that defibrotide did not show a benefit in the prophylaxis of SOS in a phase 3 clinical trial, leading to early termination due to futility [5,6].

Our retrospective database cohort study aimed to examine the preventative role of defibrotide in pediatric HSCT patients by assessing the incidence of SOS in those receiving and not receiving defibrotide prophylaxis. Additionally, we aimed to assess the impact of defibrotide prophylaxis on healthcare outcomes, including mortality, cost, and post-transplant complications.

## 2. Materials and Methods

### 2.1. Study Design

We performed a retrospective cohort study including pediatric patients aged 0–18 years of age with data stored in the Pediatric Health Information System (PHIS) database who received a HSCT between 1 January 2016 and 31 December 2022 and were classified as high-risk or very high-risk for development of SOS.

### 2.2. Data Retrieval

Demographic data and outcomes data were obtained through the PHIS database. Demographic data included age, gender, race, and type of transplant. Outcomes data included details of the transplant course to assess incidence of SOS, hospital length of stay, ICU admission, cost of admission, and overall mortality. Risk of developing SOS was assessed retrospectively with ICD-10 codes based on criteria from the HARMONY trial (Table S2). High-risk patients were defined by the HARMONY trial as containing factors such as prior HSCT, active viral infection, or advanced disease status, while very high-risk patients had multiple such factors or severe baseline liver dysfunction, as shown in Supplemental Table S1. The incidence of SOS was determined by filing of the ICD-10 code K76.5 [hepatic veno-occlusive disease] [7]. This code captures the clinical diagnosis of SOS as documented by treating physicians; however, the specific diagnostic criteria applied at each center could not be verified from the dataset. This heterogeneity in diagnostic criteria may have influenced the reported incidence of SOS across centers. Patients were divided into two cohorts for further analysis: those who received defibrotide prophylaxis, defined as patients who received defibrotide prophylaxis starting on or before day +3 post-transplant, and those who did not receive defibrotide prophylaxis. The no-prophylactic defibrotide group included patients who did not receive defibrotide prophylaxis; however, therapeutic defibrotide was administered if SOS was diagnosed following HSCT. Given the retrospective structure of the dataset and absence of reliable time-to-event information, time-to-event analyses were not performed. SOS incidence was therefore defined as the proportion of encounters complicated by SOS, rather than cumulative incidence with competing risks. Consequently, mortality was assessed separately as an outcome at discharge and not treated as a competing event.

### 2.3. Statistical Analysis

To account for multiple encounters from the same patient, mixed-effects regression models with patients as a random intercept were used for statistical analysis. The model included prophylaxis status as the primary independent variable; analyses were stratified by risk group (high vs. very high). Odds ratios (OR) with 95% confidence intervals (CI) were estimated for SOS incidence. For continuous outcomes, mixed-effects linear regression was used; for binary outcomes (ICU admission, mortality, GVHD), mixed-effects logistic regression was used. A *p* < 0.05 was considered statistically significant. All analyses were conducted using STATA version 17.

## 3. Results

### 3.1. Characteristics of the Study Population

A total of 10,250 patient encounters from 8159 unique patients were included in this retrospective data analysis and were stratified into high-risk (n = 9584) and very high-risk (n = 666) groups as summarized in Table 1. 

### 3.2. SOS Incidence with Prophylactic Defibrotide Use

Overall, the SOS incidence in our study population was 8.7% ([n = 890/10,250]; 95% CI: 8.1–9.2%). Patient encounters were also classified into two cohorts: no prophylactic defibrotide use (n = 9906) and received defibrotide prophylaxis (n = 344). In both the high-risk and very high-risk groups, SOS incidence differences between the prophylaxis and non-prophylaxis groups were not statistically significant ([n = 79/302]; high-risk 26% vs. 7.1% [n = 663/9282], OR 1.457, 95% CI 0.494–4.296, *p* = 0.495) and very high-risk (31% [n = 13/42] vs. 21.6% [n = 135/624]; OR 1.081, 95% CI 0.019–60.769, *p* = 0.970). The extremely wide 95% CI for the very high-risk group reflects the small number of events in this subgroup (n = 42 prophylaxis patients) and likely quasi-complete separation in the logistic model, precluding meaningful inference about the direction of association in this subgroup. The data are consistent with both benefit and harm of prophylaxis, and no directional conclusion can be drawn. Results are shown in Table 2.

### 3.3. Clinical Outcomes with Prophylactic Defibrotide Use

**Figure 1:**
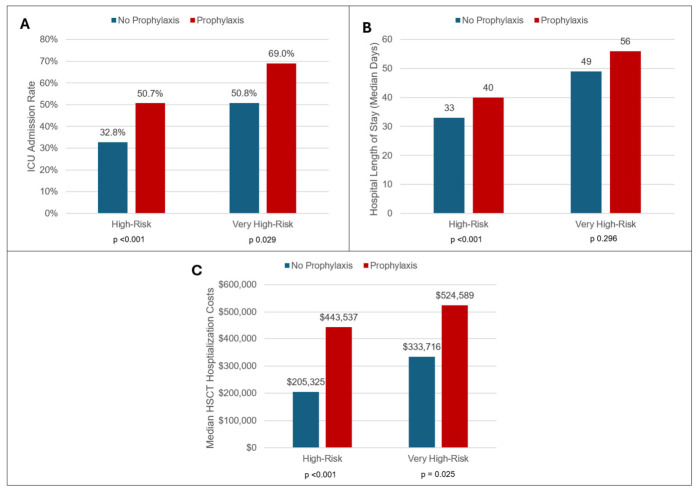
Comparison of outcomes within high-risk and very high-risk groups receiving and not receiving defibrotide prophylaxis. Patient encounters were classified into two cohorts: no prophylactic defibrotide use (n = 9906) and prophylactic defibrotide use (n = 344). (**A**) ICU admission rates, (**B**) median hospital length of stay, and (**C**) median HSCT hospitalization costs are shown for each risk group. *p* values represent comparisons between prophylaxis and no prophylaxis within each risk category. **HSCT:** hematopoietic stem cell transplant; **ICU:** intensive care unit.

The ICU admission rates were 32.8% in the no prophylaxis group and 50.7% in the prophylaxis group among high-risk patients (*p* < 0.001; Figure 1A). In the very-high risk group, ICU admission rates were 50.8% in the no prophylaxis group and 69% in the prophylaxis group (*p* = 0.029) (Figure 1A). The median hospital stay for the high-risk group was 33 days in the no prophylaxis group, compared to 40 days in the prophylaxis group (*p* < 0.001), and in the very-high risk group these were 49 and 56 days for the no prophylaxis and prophylaxis groups, respectively (*p* = 0.296) (Figure 1B). Mortality rates in the high-risk group were 3.5% vs. 7.9% when comparing the no prophylaxis and prophylaxis groups (*p* < 0.001). Mortality rates in the very high-risk group were 10.9% in patients who did not receive defibrotide prophylaxis and 23.8% in those patients who did (*p* = 0.104).

### 3.4. Cost Analysis with Prophylactic Defibrotide Use

The median HSCT hospitalization costs in the high-risk group were $205,325 [IQR $124,478–$333,419] in patients who did not receive defibrotide prophylaxis, compared with $443,537 [IQR $317,893–$649,493] in patients who received prophylaxis (*p* < 0.001). In the very high-risk group, these costs were $333,717 [IQR $217,258–$578,423] and $524,588 [IQR $365,569–$922,337] when comparing no prophylaxis to prophylaxis (*p* = 0.025) (Figure 1C). The cost differences likely reflect the higher baseline acuity and resource utilization of patients selected for prophylaxis rather than a direct cost attributable to defibrotide itself.

Busulfan is among the strongest established risk factors for SOS, and its incomplete capture represents a meaningful limitation of this study. While 2772 patients were identified as having busulfan exposure, an additional 624 patients with potential busulfan exposure could not be reliably classified due to inconsistent reporting in the PHIS database. This represents approximately 18% of expected busulfan-exposed patients who may have been misclassified. If busulfan-exposed patients were disproportionately represented in the prophylaxis group, this would further support the confounding-by-indication interpretation and may have attenuated the apparent risk differences between groups. Future studies should ensure complete capture of busulfan exposure as a critical covariate.

## 4. Discussion

Defibrotide initially was considered a potential prophylactic agent for SOS after the pivotal phase 3 trial by Corbacioglu et al. demonstrated decreased incidence of SOS in pediatric patients undergoing myeloablative HSCT with additional risk factors [5]. This open-label randomized trial, conducted exclusively in children, led to widespread adoption of defibrotide prophylaxis in pediatric transplant centers. Additional studies showed the prophylactic effect of defibrotide in adult patients who underwent allogeneic HSCT regardless of risk status [8], and a meta-analysis in 2022 showed a prophylactic effect of defibrotide regardless of age groups [risk ratio 0.30, 95% CI 0.12–0.71] [9]. However, more recent studies—including our own—call into question the utility and efficacy of this approach.

Recently, the HARMONY trial was conducted by Grupp et al., which was prematurely terminated after interim analysis due to failure to show significant benefit of defibrotide prophylaxis over best supportive care [6], which resulted in dissemination of direct health care communication in the EU to discourage the use of defibrotide as prophylaxis for VOE [10]. Our findings align with these findings, and this is the first study of this size to report on clinical outcomes of prophylactic defibrotide in a solely pediatric population. In our cohort, SOS incidence remained comparable between prophylaxis and non-prophylaxis groups across both high and very high-risk categories, highlighting the limited utility of preventative defibrotide.

The most important interpretive consideration in our study is confounding by indication. Patients who received prophylactic defibrotide were likely at higher baseline clinical risk than those who did not—this is precisely why clinicians selected them for prophylaxis. The observed associations between prophylaxis and adverse outcomes (higher SOS incidence, mortality, ICU admissions, LOS, and costs) therefore likely reflect the sicker baseline phenotype of prophylaxis recipients rather than any adverse effect of defibrotide itself. Propensity score analysis was not performed in this study; moreover, the restricted covariate set available in the PHIS database would limit the ability of such an analysis to adequately address confounding from unmeasured variables including conditioning intensity, disease stage, organ function, and prior hepatotoxic exposures. Accordingly, the observed associations should not be interpreted as evidence that defibrotide prophylaxis causes harm.

A particularly notable contribution to the evolving dialogue is the recent editorial by Corbacioglu, the lead author of the original phase 3 trial [11]. He reflects on the discordance between early-phase findings and subsequent real-world and clinical trial data, highlighting the need to reconsider this scientific question and calling for larger studies to fully explore this concept, particularly in the vulnerable pediatric population. This could be in part explained by improvement of best supportive care and early detection of signs of SOS before they meet the diagnostic criteria that lead to a subsequent decrease in incidence rate. In addition, the original study published in 2012 was conducted between 2006 and 2009, whereas the HARMONY trial was conducted between 2017 and 2020. Given that the importance of early diagnosis of SOS was more appreciated and the diagnostic guideline for SOS was updated to EBMT adult criteria in 2016 and into pediatric criteria in 2017, it is possible that populations in the original study and in the recent studies, including our study that was conducted between January 2016 and December 2022, are different. An additional consideration is the heterogeneity of SOS diagnostic criteria applied across PHIS centers during the study period. The EBMT criteria have no time limitation for SOS onset and include transfusion-refractory thrombocytopenia as a diagnostic consideration, in contrast to the Baltimore and modified Seattle criteria, which require onset within 20–21 days and mandate hyperbilirubinemia [12]. Since our study relied on ICD-10 code K76.5 for SOS identification, we cannot determine which diagnostic criteria were applied at each center, and variable adoption of the EBMT criteria may have influenced the reported SOS incidence.

In the original study, 20% of the control group, who received best supportive care, developed SOS, whereas in the HARMONY trial, 16% of the best supportive care group was recorded as SOS by the investigators at bedside. Interestingly, there is a significant discrepancy in the incidence rate of SOS recorded by the investigators and the Endpoint Adjudication Committee (EPAC) in the HARMONY trial. Incidence per investigator was 16% in the BSC group and 12% in the defibrotide-prophylaxis group; however, after EPAC review, it was 21% in the BSC group and 25% in the prophylaxis group. This could be attributed to lack of blinding due to the nature of the trial or selection criteria.

Contrary to controversy in pediatric studies, some adult studies suggest a potential benefit of defibrotide prophylaxis. A single-center adult study by Picod et al. reported an SOS incidence of just 6.3% in high-risk patients receiving prophylaxis with a favorable safety profile—substantially lower than historical controls [13]. Notably, this adult study combined defibrotide with ursodeoxycholic acid, which may offer a synergistic effect. This discrepancy raises important questions about endothelial senescence, pharmacodynamics, and transplant-related factors, including conditioning regimen and underlying diseases influencing defibrotide efficacy. This is in comparison to prior pediatric studies such as that published by Moulik et al., which demonstrated that the discontinuation of prophylactic defibrotide in pediatric patients undergoing myeloablative autologous transplants did not increase the severity or incidence of SOS [14].

While defibrotide is often reported to be well tolerated by multiple studies [15], our study found associations with increased intensive care unit admissions, longer hospital stays, and higher incidence of acute GVHD and infections in the prophylaxis group. Furthermore, patients in the high-risk category who received prophylactic defibrotide had overall higher mortality than the non-prophylactic group in our study. The very high-risk group did not show statistical significance in mortality between the prophylactic group and non-prophylactic group. These adverse outcomes contrast with the HARMONY trial, which reported similar safety profiles between defibrotide and control arms, and adult studies where bleeding-related discontinuation occurred in only a minority of cases [6,13]. Pediatric patients may be more vulnerable to severe complications, possibly due to developmental differences in vascular integrity, endothelial cell immaturity, or supportive care thresholds. Recent institutional evaluations reinforce the financial burden of routine prophylactic defibrotide. A single-center study implemented a restrictive prophylaxis policy—delaying initiation, using ideal body weight for dosing, and applying stricter inclusion criteria—without affecting SOS incidence or severity [16]. Similarly, Moulik et al. observed no increase in SOS following discontinuation of routine prophylaxis in a pediatric auto-HSCT population, including very high-risk patients [14]. Together with our findings, these results support re-examination of defibrotide prophylaxis under a close lens, especially given its high cost and uncertain benefit. Ursodiol is a more economic option for SOS prophylaxis and showed clearer benefit, but its efficacy for the high-risk population is not well established [3,17].

Based on defibrotide’s multifactorial mechanism, along with the absence of systemic anticoagulant activity, defibrotide would be an ideal candidate for prophylaxis or treatment of SOS [18]. However, whether these mechanisms are activated in the absence of active endothelial damage in vivo remains unclear. Based on our results, it appears that defibrotide’s ability to work as a preventative measure may not be as consistent as its ability to repair existing damage in the microvasculature, despite in vitro data demonstrating prophylactic benefit [19,20]. This may explain why therapeutic use of defibrotide has more concrete evidence compared to prophylactic use [8,21].

This study has several important limitations inherent to its retrospective, observational design. Propensity score analysis was not performed; residual confounding by indication from unmeasured variables (conditioning intensity, disease stage, organ function, prior hepatotoxic exposures) remains the primary interpretative limitation. Defibrotide prophylaxis was not randomly assigned, and patients receiving prophylaxis were almost certainly at higher baseline risk. No adequate adjustment for baseline severity was possible given the restraints of the administrative dataset. Residual confounding cannot be excluded, and observed association should not be interpreted as evidence of causality. Variation in SOS diagnostic criteria across centers could not be determined and may have influenced SOS incidence estimates. Additionally, given the retrospective nature of the study and limitations in data granularity, we were unable to comprehensively adjust for baseline clinical severity, including detailed organ function or transplant-specific characteristics beyond autologous versus allogeneic status.

Risk stratification adhered strictly to the validated HARMONY high-risk and very-high-risk criteria to ensure methodological consistency and comparability with existing literature. However, several critical HARMONY risk variables were incompletely captured. Prior exposure to medications associated with SOS risk (e.g., gemtuzumab ozogamicin or inotuzumab ozogamicin) could not be reliably retrieved. Liver dysfunction was identified using ICD-10 codes rather than definitive laboratory values, in contrast to prospective studies including the HARMONY trial. Busulfan exposure, one of the strongest risk factors for SOS, could not be reliably captured in 624 patients (approximately 18% of expected busulfan-exposed patients), potentially leading to a misclassification of risk status in both groups. Similarly, iron overload could not be quantified, as PHIS does not allow assessment of iron burden severity, resulting in heterogeneous classification that may attenuate risk associations.

The PHIS dataset structures data at the encounter level, limiting the ability to capture outcomes occurring after discharge. As a result, infections were only captured when they occurred during the index transplant hospitalization, and events arising in subsequent encounters could not be reliably linked to a specific transplant. Late-occurring complications after hospital discharge therefore could not be incorporated into the analysis. Events such as infections documented late in follow-up (e.g., beyond day +300 post-transplant) are unlikely to be biologically attributable to a brief prophylactic exposure in the early post-transplant period and more plausibly reflect residual confounding related to underlying disease severity, transplant complexity, or subsequent clinical course. Similarly, lack of granular timestamps precluded precise temporal alignment of complications such as viral reactivation with defibrotide exposure.

The dataset also does not contain clinical measures necessary to assess SOS severity, including bilirubin trajectory, weight gain, hepatomegaly extent, or organ dysfunction parameters. Consequently, differentiation between mild and severe SOS was not possible. Data on transplant-associated thrombotic microangiopathy, renal replacement therapy, mechanical ventilation, and other secondary procedures were not consistently available in this retrospective cohort and therefore could not be evaluated, limiting the granularity of outcome assessment beyond ICU utilization and in-hospital mortality. Finally, the inability to fully characterize disease stage, refractory or relapsed status, and other markers of advanced malignancy introduces additional residual confounding. While certain diagnostic categories were identifiable, systematic capture of all forms of advanced disease was not feasible within this administrative dataset.

This study was unable to compare severity of SOS in prophylaxis and non-prophylaxis groups due to limited data retrieval. Severity of SOS has not been compared in the original study in 2012 or the HARMONY trial, but it is possible that prophylactic defibrotide may not decrease the incidence but may decrease the severity of SOS, even though neither study shows a difference in mortality between the two groups.

Lack of standardized care regarding initiation of prophylactic defibrotide use, supportive care prior to development of SOS, and initiation of SOS treatment introduces heterogeneity between centers, limiting generalizability and complicating cross-study comparisons. Since the PHIS dataset does not permit reliable differentiation between prophylactic and therapeutic intention of defibrotide administration, timing relative to transplant was used as a proxy. SOS has a peak incidence at 7–21 days after transplant, but some develop SOS as early as within a couple of days after transplantation. There could be misclassification of prophylactic vs. therapeutic defibrotide use, and it would be indistinguishable. 

Future work should focus on identifying molecular predictors of SOS and integrating them into risk-adapted prevention strategies. Randomized controlled trials are urgently needed to assess optimal timing, duration, and patient selection for preventative therapies. Parallel investigation into adjunctive or alternative agents is warranted.

## 5. Conclusions

Our study, alongside the HARMONY trial, registry studies, and recent institutional analyses, contributes to a growing body of evidence warranting re-evaluation of routine defibrotide prophylaxis in pediatric HSCT. Despite its theoretical benefits, prophylactic defibrotide use was not associated with reduced SOS incidence in our cohort and was associated with increased resource utilization and adverse events. However, these observed associations likely reflect confounding by indication—the preferential use of prophylaxis in clinically sicker patients—rather than a direct harmful effect of defibrotide. The recent EBMT consensus guidelines continue to support consideration of defibrotide prophylaxis in selected high-risk pediatric patients using a weighted scoring system, reflecting the ongoing clinical equipoise [22]. These findings contribute hypothesis-generating evidence supporting reevaluation of SOS prophylaxis practice and underscore the need for prospective clinical trials with appropriate confounding adjustment to definitively determine the role of prophylactic defibrotide in SOS prevention.

## Figures and Tables

**Table 1:** Patient demographics.

	High-Risk (n = 9584)	Very High-Risk (n = 666)
Age, median (IQR)	6 (2–13)	4 (1–10)
Gender, n, (%)		
Female	3967 (41.4%)	232 (34.8%)
Male	5617 (58.6%)	433 (65%)
NR		1 (0.002%)
Race, n (%)		
White	5719 (59.7%)	366 (55%)
Black	1356 (14.1%)	84 (12.6%)
Asian	386 (4%)	35 (5.3%)
Other	1514 (15.8%)	125 (18.8%)
Multiracial	214 (2.2%)	16 (2.4%)
NR	395 (4.1%)	40 (6%)
Transplant type		
Allogeneic	5614 (58.6%)	567 (85.1%)
Autologous	3970 (41.4%)	99 (14.9%)
Defibrotide (DF) prophylaxis, n (%)		
No DF prophylaxis	9282 (96.8%)	624 (93.7%)
DF prophylaxis	302 (3.2%)	42 (6.3%)
Incidence of SOS, n (%)	742 (7.7%)	148 (22.2%)

IQR: interquartile range; NR: not reported; SOS: sinusoidal obstruction syndrome.

**Table 2:** Incidence of SOS based on risk group and prophylaxis cohort.

	No Prophylaxis (n = 9906)	Prophylaxis (n = 344)	*p*-Value
High-risk (n = 9584)	7.1%	26%	*p* = 0.495 OR: 1.457 (95% CI: 0.494–4.296)
Very high-risk (n = 666)	21.6%	31%	*p* = 0.970 OR: 1.081 (95% CI: 0.019–60.769)

## Data Availability

The original contributions presented in this study are included in the article/Supplementary Material. Further inquiries can be directed to the corresponding author.

## Supplementary Materials

The following supporting information can be downloaded at: https://www.mdpi.com/article/10.3390/jpm16070368/s1, Table S1: High-Risk and Very High-Risk Classification Criteria and ICD-10 Codes; Table S2: Additional Outcomes in High-Risk and Very High-Risk Groups Receiving and Not Receiving Defibrotide Prophylaxis.
