# Supplementary material for: Evaluation of Prophylactic Defibrotide Use in Pediatric Hematopoietic Stem Cell Transplant Recipients: A Multicenter Retrospective Cohort Study
Source: J Pers Med. 2026 Jul 7;16(7):368. doi: 10.3390/jpm16070368 (PMC13412807; doi:10.3390/jpm16070368)
Supplement: Supplementary file 1 [file jpm-16-00368-s001.zip › jpm-4305615-supplementary.pdf]

Supplemental Table S1. High-Risk and Very High-Risk Classification Criteria and ICD-10 Codes.

High-Risk	Very High-Risk
1. Advanced stage neuroblastoma (C7490) n = 825<br>2. Transaminases >2.5x ULN (R945) n = 30<br>3. Serum bilirubin >1.5x ULN (R17) n = 163<br>4. Cirrhosis (K746) n = 0<br>5. Active viral hepatitis (B159) n = 2<br>6. Abdominal or hepatic irradiation (Z510) n = 33<br>7. Iron overload (E8311) n = 0<br>8. Busulfan n = 2772<br>9. Hepatotoxic medication use n = 10142	Diagnosis of any of the following as identified by ICD-10 code:<br>1. Osteopetrosis (Q782) n = 36<br>2. Primary immunodeficiency (D80) n = 0<br>3. Primary HLH (D761) n = 269<br>4. High-risk thalassemia (D5609) n = 0<br>5. Hepatomegaly (R160) n = 313<br>6. Adrenoleukodystrophy (E71529-E71520) n = 62

HLH: hemophagocytic lymphohistiocytosis, ULN: upper limit of normal, n: number of patients.

Supplemental Table S2. Additional Outcomes in High-Risk and Very High-Risk Groups Receiving and Not Receiving Defibrotide Prophylaxis.

	**No Prophylaxis (n=9,906)**	**Prophylaxis (n=344)**	**p-value**
Acute GVHD (%)			
High-Risk	7.7%	11.9%	0.872
Very High-Risk	13.1%	7.1%	0.930
Chronic GVHD (%)			
High-Risk	0.3%	0.7%	Not performed
Very High-Risk	0.8%	0%	Not performed
CMV Infection (%)			
High-Risk	7.6%	12.6%	0.502
Very High-Risk	9.5%	14.3%	0.916
EBV Infection (%)			
High-Risk	0.1%	0%	Not performed
Very High-Risk	0.6%	0%	Not performed
ADV Infection (%)			
High-Risk	0.8%	0.3%	Not performed
Very High-Risk	2.2%	0%	Not performed
Sepsis (%)			
High-Risk	11.7%	15.9%	0.027
Very High-Risk	24.8%	26.2%	0.772

ADV: adenovirus; CMV: cytomegalovirus; EBV: Epstein-Barr virus; GVHD: graft-versus-host disease.
